# Host/microbiota interactions in health and diseases—Time for mucosal microbiology!

**DOI:** 10.1038/s41385-021-00383-w

**Published:** 2021-03-26

**Authors:** Noëmie Daniel, Emelyne Lécuyer, Benoit Chassaing

**Affiliations:** 1 INSERM U1016, team “Mucosal microbiota in chronic inflammatory diseases”, CNRS UMR 8104, Université de Paris, Paris, France; 2 Microenvironment & Immunity Unit, Pasteur Institute, INSERM U1224, Paris, France

## Abstract

During the last 20 years, a new field of research delineating the importance of the microbiota in health and diseases has emerged. Inappropriate host-microbiota interactions have been shown to trigger a wide range of chronic inflammatory diseases, and defining the exact mechanisms behind perturbations of such relationship, as well as ways by which these disturbances can lead to disease states, both remain to be fully elucidated. The mucosa-associated microbiota constitutes a recently studied microbial population closely linked with the promotion of chronic intestinal inflammation and associated disease states. This review will highlight seminal works that have brought into light the importance of the mucosa-associated microbiota in health and diseases, emphasizing the challenges and promises of expending the mucosal microbiology field of research.

## Introduction

The intestinal microbiota is a vast and complex community of microorganisms inhabiting the gastrointestinal tract, encompassing 10^13^ bacteria per intestine and about 100–500 different species per individual.^1,2^ Among its functions in numerous aspects of host physiology, the gut microbiota is essential to promote maturation of the intestinal immune system as well as digestion by providing extraction of calories and nutrients that would otherwise be excreted in feces. This beneficial equilibrium between the host and its microbiota, or symbiosis, can also turn detrimental and participate in the development and/or the worsening of chronic inflammatory diseases.^3,4^ Hence, efforts have been made toward characterization of gut microbiota composition and function in health and diseases. Numerous studies have reported microbiota alterations in both preclinical and clinical models of chronic inflammatory diseases. Although those alterations may, in part, be driven by the disease state, recent works have highlighted that an altered microbiota can also play a central role in driving the disease itself, with the observation that disease can be transferred to germ-free mice through microbiota transplantation.^5^

Altogether, these findings suggest that the intestinal microbiota holds the potential for innovative therapeutic approaches, where microbiota modulations through fecal microbiota transplantation, diet, prebiotic or probiotic can be used to treat and/or prevent diseases. However, except for recurring *Clostridium difficile* infections, none of these approaches have demonstrated efficiency to restore host-microbiota homeostasis in chronic inflammatory disease patients. First, a great resilience is observed regarding human microbiota composition, so that its modulation is difficult to achieve and often only transient.^6^ Second, high interindividual variations, driven by genetic and environmental factors, are observed in human microbiota composition, suggesting the need for microbiota-based patient stratifications.^7^ Third, more work is still required to identify select microbiota alterations that are playing a role in disease progression, instead of simply being associated with it. Finally, the vast majority of the research performed on the intestinal microbiota is focusing on stool samples, while accumulating data demonstrate the importance of mucosa-associated microbiota in health and diseases. While some reviews previously elegantly described the role of host/microbiota interactions, they highlighted that these interactions are far from being characterized at the mucosal interface.^8–11^ Hence, we will highlight here several works that demonstrate the importance of the mucosa-associated microbiota, and we will discuss the promises of expanding the mucosal microbiology field of research.

## Intestinal microbiota composition—3 dimensions matter

### Longitudinal axis

As previously summarized by Donaldson et al., the lower gastrointestinal tract (i.e., the small intestine, the cecum, and the colon) harbors different habitats populated by specific bacterial communities, in a way that several microbial communities can be identified within the same individual and along a longitudinal axis.^12^ Each of these intestinal compartments is characterized by specific physiological, chemical, nutritional and immune conditions, which altogether shape region-specific communities. For example, and following the discovery of *Helicobacter pylori*, a limited microbiota comprising few genera (*Propionibacterium*, *Lactobacillus*, *Streptococcus* and *Staphylococcus*) was found in the stomach, while this organ was previously regarded as sterile.^13^ The small intestine harbors *Lactobacillaceae* (Firmicutes) and *Enterobacteriaceae* (Proteobacteria) facultative anaerobe families able to tolerate the acidic environment, as well as high levels of oxygen and antimicrobial molecules (e.g., bile acids).^14,15^ Finally, the cecum and the colon are mainly intended for complex carbohydrate fermentation and contained a denser and more diversified population, composed of the following dominant bacterial phyla: Bacteroidetes, Firmicutes, Actinobacteria, Proteobacteria, and Verrucomicrobia. This final part of the gastrointestinal tract is harboring what is considered the densest bacterial community on earth.

### Transversal axis

The impact of the transversal axis on microbiota composition has been recently questioned. Work from several research teams revealed that several microenvironments exist and influence radial spatial distribution of bacterial communities.

### From the lumen to the epithelium

It was for example demonstrated, in several mammal models (murine, swine, macaque or human), that the luminal and the mucosa-associated microbiota are significantly different in terms of composition (Table 1).^16–21^ From 1965 to 1972, a series of histological studies reported that bacteria embedded in the mucous layers of the epithelium had a distinct fusiform shape compared to those present in the lumen.^16–18^ Novel approaches, such as laser capture microdissection (LCM), further attempted to understand the impact of the transversal axis on microbiota composition. In the mouse ascending colon, Nava et al. notably demonstrated that the mucosa constitutes a specialized niche enriched in Firmicutes phylum (*Lachnospiraceae*, *Ruminococcaceae* families), while the luminal region is colonized by *Bacteroidaceae*, *Enterococcaceae*, and *Lactobacillaceae* families.^22^ In another interesting study, Liu et al. used the in vitro Twin Simulator of the Human Intestinal Microbial Ecosystem (TWIN-SHIME) model to compare luminal and mucosal communities tropism. With such an approach, unique mucosa-associated and lumen-associated microbial communities were observed in each of the colonic regions tested (ascending, transverse, and descending).^23^ While multiple studies demonstrated the existence of a transversal axis influencing microbiota composition, crypts- and inner mucus-associated microbiota have recently gained a lot of attention.

**Table 1:** Studies demonstrating the importance of the mucosa-associated microbiota in health and diseases.

	Model	Main findings	Ref.
Steady state	Mouse	- Discovery of the “autochthonous bacterial flora”. Bacterial populations are different between the epithelium and the lumen.	^16–18^
	Mouse	- Mucosa-associated microbiota is different from the luminal microbiota: *Actinobacteria* is more abundant in the lumen, while *Acidobacteria*, *Deferribacteres* and *Proteobacteria* are enriched in the mucosa.	^19^
	Macaque	- The lumen harbors obligate anaerobes, while mucosa-associated microbiota is enriched in oxygen-tolerant bacteria.	^20^
	Pig	- Firmicutes are more abundant in the digesta, while Proteobacteria and Bacteroidetes are enriched in the mucosa.	^21^
	Mouse	- In the ascending colon, the digesta is enriched in *Bacteroidaceae*, *Enterococcaceae* and *Lactobacillaceae*, while crypts constitute special niches sheltering *Lachnospiraceae* and *Ruminococcaceae* families.	^22^
	Mouse & Human	- *Lachnospiraceae* and *Ruminococcaceae* are present at the same relative abundance in human biopsies than in the interfold region in mice. Some bacteria are species and region dependent: *Faecalibacterium* or *Subdoligranulum* are only detected in human biopsies, while *Marvinbryantia* is inherent to mice and *Butyrivibrio* only localized in mice interfold region.	^187^
	Mouse	- Identification of the colonic crypt-associated microbiota (CSCM). Lumen is richer in Firmicutes while CSCM is composed of Proteobacteria, aerobic genera (*Burkholderiales*, *Xanthomonadale*).	^25^
	Mouse & Human	*- Acinetobacter* genus has a particular tropism for the crypt environment	^25,26^
	Mouse	- SFB is a commensal bacterium adherent to the ileal epithelium and playing a symbiotic role.	^89,94,95,99,100,102^
	Mouse	- *Citrobacter rodentium* is able to attach to the intestinal epithelium and grow through the hydrogen peroxide (H_2_O_2_) respiration, with a central role played by the NAPDH oxidase NOX1.	^126^
IBD/IBS	Human	- Colonic biopsies from IBD patients are characterized by increased bacterial encroachment and paracellular and vascular permeability.	^56^
	Human	*- E. coli*, *Clostridium* and *Bacteroides* are enriched in the mucosa of IBD patients compared to controls, while *Bifidobacteria* is decreased. In active UC disease, *E. Coli* and *Bacteroides* are found in the lamina propria.	^58^
	Human	- Reduced bacterial diversity is found in the mucosa-associated microbiota of IBD patients compared to controls.	^59,61,62^
	Human	- Crohn’s disease recurrence following a partial bowel resection can be predicted by analysis of the ileal mucosa-associated microbiota (role of *Gammaproteobacteria*, *Ruminococcus gnavus* and *Corynebacterium*)	^72^
	Human	*- Brachyspi*ra is found in the mucosa-associated microbiota of 30–40% IBS patients.	^73^
	Human & Mouse	*- Faecalibacterium prausnitzii* is reduced in IBD patients. Multiple strains, as well as *Faecalibacterium prausnitzii* supernatant, polymeric extracellular matrix and a purified protein are sufficient to decrease intestinal inflammation in animal models.	^62,188–190^
	Human	- Adherent-Invasive *Escherichia coli* (AIEC) pathobionts (*Enterobacteriaceae)* are found in the mucosa of 30–40% IBD patients, compared to 5–10% in healthy individuals.	^64,66^
	Mouse	- AIEC are flagellated and express a mucinase, which enhance their ability to adhere to and penetrate the intestinal mucus barrier.	^71,191^
	Mouse	- Emulsifier-induced colitis is associated with microbiota encroachment, altered microbiota composition and increased pro-inflammatory potential.	^50^
	Mouse	- Flagellin immunization increase host-microbiota distance and protect against colitis and obesity.	^184^
Diabetes	Mouse	- Emulsifier-induced metabolic syndrome is associated with microbiota encroachment, altered microbiota composition and increased pro-inflammatory potential.	^50^
	Mouse	- A complex microbiota containing specific species infiltrating the mucus layer is required for the detrimental effects of emulsifiers.	^144^
	Human	- Microbiota encroachment is a feature of metabolic disease, particularly hyperglycemia, in humans.	^51^
	Mouse	- Western diet (WD) affects the growth rate and penetrability of the colonic mucus layer.	^131^
		- WD-associated deleterious effects are reversed by soluble fiber consumption.	^131,135,140,141^
	Mouse & Human	*- Akkermansia muciniphila* is a commensal bacterium whose administration strengthens intestinal epithelium integrity and reverse metabolic disorders.	^149,157,161^
Colorectal cancer	Human	*- Fusobacterium* and *Bacteroides fragilis* are enriched in biopsies from right-side tumors, while *Parvimonas micro* is enriched in biopsies from left-side tumors.	^26^
	Human	- Identification of *Fusobacterium nucleatum* in biopsies of CRC patients.	^55,80^
	Human	- Identification of Enterotoxigenic *E. coli* and *Bacteroides fragilis* (ETBF) in colonic mucosa-associated biofilm from familial adenomatous polyposis patients.	^81^

### Crypt-associated microbiota

While the distribution of the gut microbiota along a radial axis was unraveled, pioneering work from Sansonetti’s team,^24^ combining Whartin—Starry (silver/nitrate) staining and FISH with 16S rRNA probes, established the existence of a “crypt-specific core microbiota” (CSCM).^25^ This CSCM was composed of limited bacterial members, which exclusively colonized the cecum and the proximal part of the colon, while being absent in the duodenum, jejunum and distal colon. Using microdissection and sequencing approaches, Firmicutes and Proteobacteria phyla were observed to be predominant in luminal crypts, while Bacteroidetes phylum was poorly represented.^25^ Interestingly, aerobic bacteria belonging to the *Burkholderiales* or *Xanthomonadales* groups dominated the CSCM, suggesting an oxygen-enrich environment close to the crypt. Follow-up studies demonstrated that *Acinetobacter* genera (Proteobacteria phylum, *Moraxellaceae* family) is more abundant in the crypts compared to the luminal environment, in both mice and humans.^25,26^ Besides *Acinetobacter*, the same group demonstrated that *Delftia tsuruhatensis* and *Stenotrophomonas maltophilia* are also important members of the CSCM.^27^ Following monocolonization of germ-free mice, these bacteria were detected in the colonic crypts, while the small intestinal crypt remained sterile.^27^ Highlighting their impact on intestinal homeostasis, the study of these CSCM members revealed their ability to decrease proliferation of epithelial cells, hence suggesting a central role for this community in intestinal homeostasis.^27^ Using an organoids approach, it was reported that, compared to a sonicated sample of Gram-positive bacteria grown in vitro, purified lipopolysaccharide (LPS) from CSCM-associated species led to organoid hypotrophy and stimulation of goblet cell differentiation that associated with *IL-33* and *Muc2* upregulation.^27^ Hence, this research elegantly demonstrated that the intestinal crypt environment harbors a unique microbial community of central importance for the mutualistic relationship between the host and its microbiota.

### Inner mucus layer-associated microbiota

Besides crypt-associated microbiota, the role of the inner-mucus layer-associated microbiota in health and diseases has recently gained attention. While studies focusing on mucosa-associated microbiota are using crude extract from colonic biopsies, encompassing outer mucus-, inner mucus-, epithelium- and crypt-associated bacteria,^17,19,22,23,25,26^ more targeted approaches on microbiota members specifically colonizing the inner-mucus layer have demonstrated their association with detrimental outcomes in preclinical and clinical models. The importance of this mucus-associated microbial community in health and diseases will be described below (Chapter “*Why is mucosa-associated microbiota important?”*)

## Time also matters

### From birth to old age

Intestinal colonization by microorganisms starts at birth and undergoes significant changes during the first years of life.^28,29^ Fecal analyses have for example demonstrated that the *Bacilli* class is dominant after birth and subsequently decreases during the first 2 months, with concomitant increases in *Clostridia* and *Gammaproteobacteria*.^30^ Moreover, various perinatal parameters, encompassing health status of the mother, mode of delivery, antibiotic usage and type of feeding, are influencing bacterial colonization of the infant gut.^31–33^ Bergström et al. reported that the intestinal microbiota is subjected to significant changes between 9 and 18 months of age, with cessation of breastfeeding and introduction of solid food being essential factors impacting its composition.^34^ Interestingly, such microbiota shifts around the weaning period are associated with profound impacts on the host immune system maturation and function.^35,36^ Al Nabhani et al. elegantly demonstrated that the intestinal immune system undergoes a strong “weaning reaction” in young mice during which the intestinal microbiota is a key actor for the development of a balanced immune system.^37,38^ Importantly, such weaning reaction is central to protect against numerous inflammatory diseases later in life, in part through the induction of RORγt + regulatory T cells.^37,38^

In adults, a core microbiota of around 40 species that account for more than 75% of the community can be detected per subject over a 1-year period, suggesting that some microbiota members may be residents for decades.^39,40^ In the elderly population, fecal microbiota appears enriched in *Enterobacteriaceae* and depleted in *Clostridium* cluster IV and XIVa as well as *Bifidobacterium*.^41^ While some links have been established between alterations in the intestinal microbiota composition and type 2 diabetes (T2D), cancer (see below) and Alzheimer’s disease,^42^ the exact impact of microbiota evolution on senescence and age-associated diseases remains to be fully elucidated.^43,44^

### Circadian oscillations

The intestinal microbiota is also subjected to circadian oscillations. In humans, Kaczmarek et al. demonstrated that the intestinal microbiota composition fluctuates during the day.^45^ Moreover, elegant mouse studies bring into light the close relationship between microbiota and the light/dark cycle, as well reviewed by Parkar et al.^46^ Importantly, perturbations of these circadian oscillations can trigger microbiota perturbations and intestinal barrier dysfunction.^47,48^ Moreover, circadian oscillations of serum metabolites are a microbiota-dependent process, and the intestinal microbiota has been observed as a central actor in modulating the circadian liver transcriptome and detoxification ability.^49^ Altogether, these data demonstrate that numerous spatial and temporal factors act in concert to finely regulate the intestinal microbiota composition and function.

## Why is the mucosa-associated microbiota important?

### Metabolic disorders

**Fig. 1:**
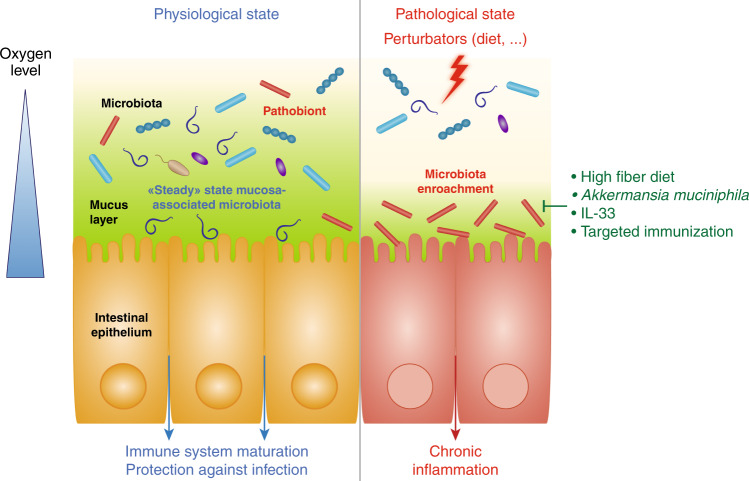
Host/microbiota interaction at the mucosal surface. At steady state (left part), the mucus layer keeps the bacterial community at a safe distance, while select symbionts favor maturation of the mucosal immune system by interacting with the host epithelium. In response to various stressors (right part), the mucus layer is altered in a way that leads to microbiota encroachment and chronic inflammatory diseases. Such altered host/microbiota relationship can be reversed by fiber-rich diet consumption, *Akkermansia muciniphila* administration or targeted mucosal immunization.

The potential role played by mucosa-associated microbiota in chronic inflammatory diseases was highlighted by multiple models of inflammation describing an “aggressive” microbiota able to penetrate the normally sterile inner mucus layer (Fig. 1). For example, consumption of synthetic dietary emulsifiers, carboxymethylcellulose (CMC) and polysorbate 80 (P80), altered mucosa-associated microbiota composition and function, leading to low-grade intestinal inflammation and metabolic disorders in mice^50^ (*cf*. details below). Importantly, it was also previously reported that microbiota encroachment is a feature of metabolic deregulations in humans, with the observation that microbiota-epithelium distance is inversely correlated with body mass index, fasting glucose levels, and hemoglobin A1C.^51^ Moreover, pioneering work by Cani et al. elegantly demonstrated that endotoxemia, referring to the elevation in circulating LPS, is associated with the promotion of metabolic disorders such as T2D and obesity.^52^ Such endotoxemia can occur following microbiota disturbance and/or increase in intestinal permeability, for example induced by a high-fat diet (HFD) regimen.^53^ Hence, these studies further illustrated the need for a well-controlled host/microbiota interaction at the mucosal surface.

### Inflammatory bowel diseases (IBD) and irritable bowel syndrome (IBS)

IBD include Crohn’s disease (CD) and ulcerative colitis (UC) that are chronic idiopathic disorders causing inflammation in the gastrointestinal tract.^54^ The etiology of these diseases has been linked to genetic factors and aberrant immune response to the gut microbiota.^55–58^ Decreased microbiota diversity was repeatedly found in fecal^59,60^ and mucosal^61,62^ samples from IBD patients, with depletion in anaerobic bacteria such as *Bacteroides*, *Eubacterium* or *Lactobacillus*.^61^ For example, lower abundance of *Faecalibacterium prausnitzii* (Firmicutes phylum) was observed in IBD patients^60,62^ in a way that associates with a higher risk of relapse.^62,63^ Furthermore, investigating microbiota organization along the transversal intestinal axis appears of central importance in IBD, with the presence of adherent and invasive microbiota members. Members of the Enterobacteriaceae family, such as the adherent invasive *Escherichia coli* (AIEC) pathovar,^64,65^ have an increased prevalence in the mucosa of IBD patients compared with healthy controls.^66–69^ AIEC pathobionts are flagellated and express multiple virulence factors with unique regulation of their expression, such as the Vat mucinase that allows mucins degradation.^70^ Altogether, these factors enhance AIEC’s ability to penetrate the mucus layer and to adhere to and invade intestinal epithelial cells in a way that promotes chronic intestinal inflammation.^71^ Ongoing studies suggest that fecal screening of AIEC bacteria fails to properly identify people carrying such bacteria in their intestinal mucosa, highlighting the importance to characterize this specific microbial population in IBD patients. Supporting this concept, a recent study demonstrated that CD relapse following partial bowel resection can be predicted by mucosa-associated microbiota composition in the ileum.^72^

IBS is a gastrointestinal disorder syndrome whose etiology remains unclear. The rectal mucosa-associated microbiota has been proposed as a potential predictor of small intestinal overgrowth, a common feature of IBS.^73^ Jabbar et al. recently analyzed mucus from sigmoid colon biopsies through metaproteomic approaches. They identified *Brachyspira* in 30–40% of IBS patients, suggesting a role for this bacterium in IBS, and further highlighting the importance of the mucosal microbiota in gastrointestinal disorders.^74^

### Colorectal cancer

Colorectal cancer (CRC) is the third most prevalent cancer worldwide and is associated with a high lethality rate.^75^ Alterations of fecal and tumor-associated microbiota composition have been described.^76,77^ Some bacterial strains have been associated with susceptibility to CRC and have focused particular attention during the past decade, such as *Fusobacterium nucleatum*, enterotoxigenic *Bacteroides fragilis* (ETBF) and *Escherichia coli* expressing the polyketide synthase genomic island that enables colibactin expression.^55,78–82^ Efforts toward identification of CRC-associated microbiota have also highlighted the importance of mucosa-associated microbiota.^26,81,82–87^ For example, Saffarian et al. characterized crypt-associated and mucosal communities in CRC. They identified Bacteroidetes, Firmicutes and Proteobacteria in murine and human colonic crypts, as well as differential microbial signatures according to the colonic area. For example, *Fusobacterium* and *Bacteroides fragilis* were enriched in right-sided tumors, while *Parvimonas micro* abundance was elevated in left-sided tumors.^26^ However, the exact role played by such alterations in tumorigenesis remains to be fully characterized.

## Are mucosa-associated bacteria always bad?

While, as highlighted above, mucosa-associated microbiota is involved in various disease states, it also appears as a critical factor influencing maturation of the intestinal immune system.

### Segmented filamentous bacteria

The best example illustrating this concept is the work on Segmented Filamentous Bacteria (SFB), which is able to stimulate both innate and adaptive immune responses, notably Th17 (Fig. 1). This bacterium is adhering to the epithelium in the terminal region of the small intestine. It harbors a nipple-like appendage inserted into the epithelium, especially in the follicle-associated epithelium of Peyer’s patches,^88^ which forms an attachment site with pronounced actin rearrangements.^89–91^ While adherence to the epithelium is a hallmark of enteropathogens, SFB are autochthonous bacteria found to play an essential role for the host immune system. SFB colonization is observed after weaning and for 2 months before declining in mice, while in humans, its presence is detected in the first 2 years of life.^92–95^ Studies using SFB-monocolonized mice showed that SFB stimulate mouse intestinal and peripheral IgA responses with a potent activation of Peyer’s patches germinal centers, gut-associated lymphoid structures required for SFB-mediated intestinal IgA responses.^96–98^ Gaboriau-Routhiau et al. demonstrated that SFB monocolonisation is sufficient to recapitulate both innate and adaptive immune priming with strong Th1, Th2, Th17 and Treg responses compared to germ-free mice,^99^ and Ivanov et al. also demonstrated the central role played by SFB in Th17 responses. Associated with its strong immunostimulatory capacities, SFB has also been described to contribute to colonization resistance against several enteric pathogens such as *Citrobacter rodentium* and *Salmonella enteritidis*.^100,101^ More recently, Shi et al. reported that SFB colonization is sufficient to protect mice against rotavirus infection.^102^ Hence, while mucosa-associated bacteria can promote chronic inflammatory diseases, data on SFB demonstrate that this microbial community is also involved in central aspects of symbiosis. Modulation/inhibition of mucosa-associated bacteria should therefore be performed with extreme caution, and recent success in in vitro culture of SFB should bring important knowledge on such duality.^103^

### Bacteroides fragilis

Works from Mazmanian et al. bring into light the central role of *Bacteroides fragilis* at the mucosal surface. After observing that this bacterium can bind to the mucus layer,^104^ they demonstrated that *B. fragilis* possesses a unique genetic locus of commensal colonization factors, referred to as the CCF system, enabling this bacterium to reside within the mucosal surface.^105^ It was also observed that, at steady state, the immune system tolerates *B. fragilis* despite its mucosal localization, with a mechanism that involves intestinal IgA.^106^ Moreover, *B. fragilis* is an obligate anaerobe but can nonetheless tolerate the oxidative stress caused by the epithelium through the alkyl hydroperoxide reductase, suggesting that this bacterium is well equipped for a mucosal niche colonization.^107^ Importantly, through the expression of its polysaccharide (PSA), *B. fragilis* can modulate T cell responses and cytokine production.^108^ Mechanistically, *B. fragilis* delivers its PSA to dendritic cells through outer membrane vesicles recognized by TLR2, which subsequently trigger immunomodulatory effects.^109^ In several models, including colitis, CRC and viral encephalitis, this bacterium was observed to be highly protective, further demonstrating the ability of select mucus-associated microbiota members to promote health.^110–112^

## Regulation of the mucosa-associated microbiota

### The oxygen hypothesis

The gut microbiota is influenced by several environmental factors, including oxygen concentration. As mentioned previously, the CSCM niche is mainly dominated by aerobic genera (e.g., *Acinetobacter*, strictly aerobic), suggesting an oxygen-enriched environment in the proximity of the epithelium.^113^ Combining an intraluminal probe with a phosphorescent quenching method, Albenberg et al. assessed oxygen distribution along the radial axis of the mouse intestine and demonstrated that it diffuses from epithelial cells to the lumen.^114^ In humans, the analysis of mucosal biopsies showed that adherent bacteria (Proteobacteria, Actinobacteria) were more aerotolerant than luminal bacteria and preferentially metabolize proteins instead of carbohydrates as substrates. Moreover, fecal microbiota composition is altered following hyperbaric oxygen therapy in mice, suggesting the impact of oxygen on the transversal microbiota compartmentalization.^114^

Several observations indicate that alteration in this radial oxygen distribution may lead to opportunistic pathogens invasion and disease development.^113,115,116^ Expansion of the aerobic zone is notably suspected of playing a role in inflammatory bowel diseases (IBDs). Indeed, IBD-associated dysbiosis is characterized by a decreased proportion of strict anaerobes (*Faecalibacterium prausnitzii*) in combination with the overgrowth of facultative aerobes, particularly the *Enterobacteriaceae* family (AIEC).^60–62,66,67,69,117,118^ Rigottier-Gois assimilated this dysbiosis to dysanaerobiosis, creating a favorable environment for the growth and invasion of aerotolerant bacteria that can enhance inflammation.^113^

The oxygen hypothesis was further consolidated by works from Baümler et al. exploring mechanisms lowering colonization resistance against *Enterobacteriaceae*, which demonstrated that antibiotic treatment promotes the shift from anaerobe to aerobe bacteria through various mechanisms.^116^ Streptomycin was reported to disrupt gut microbiota composition, depleting *Clostridia* class.^116,119,120^ Yet, *Clostridia* are important producers of short-chain fatty acids, such as butyrate, which is used as an energy source by mature colonocytes.^121,122^ Butyrate metabolization requires substantial quantities of oxygen, promoting a hypoxic environment close to the epithelium and avoiding aerobic bacteria colonization.^123,124^ In the absence of butyrate, colonocytes switch to glucose fermentation to obtain ATP. Hence, streptomycin, by reducing *Clostridia*, indirectly favors *Enterobacteriaceae* bacteria by generating an aerobic niche.^116,120^ In addition, streptomycin was reported to promote synthesis of the inducible nitric oxide synthase and subsequent production of nitric oxide, a reactive nitrogen species suggested to catalyze monosaccharide oxidation, leading to an increase in resources critical for pathogens (e.g., glucarate and galactarate that constitute a nutrient niche for species like *E. coli* and *S. typhimurium*).^125^ These studies elegantly indicate that controlling oxygen concentration at the epithelium surface may represent a possible mechanism to shape bacterial colonization of this specific intestinal niche. Interestingly, recent work from Bäumler’s team demonstrated that *Citrobacter rodentium* pathogen is able to intimately attach to the intestinal epithelium and grow through hydrogen peroxide (H_2_O_2_) respiration in the non-inflamed gut, with a central role played by the NAPDH oxidase NOX1. These data suggest that H_2_O_2_ can also be an important player in the regulation of the mucosa-associated microbiota.^126^

### Dietary factors

Among factors modulating bacterial colonization at the mucosal side, diet is certainly one of the most important. As highlighted below, several dietary factors may alter mucosa-associated microbiota in a way that promotes chronic intestinal inflammation.

### Western diet

Western Diet (WD) is characterized by an increased fat intake, refined sugars/sweeteners and animal proteins, and a decreased consumption of fruits, vegetables, and whole grains.^127^ WD consumption is strongly associated with metabolic disorders, such as obesity, T2D, and non-alcoholic liver disease. Over the last decades, the role of the host/microbiota relationship was demonstrated in several WD-induced pathologies (reviewed in^128,129^). It was for example shown that HFD shifts commensal bacterial composition, increasing Proteobacteria, while decreasing Bacteroidetes relative abundance.^130^ Interestingly, Schroeder et al. reported that WD affects the growth rate and penetrability of the colonic mucus layer compared to a control fiber-rich diet.^131^ Moreover, WD-fed mice displayed reduced inner mucus layer thickness and slower mucus growth.^131^ They also observed increased mucus penetrability of pathogens, as well as goblet cells hypertrophy and Muc2 and Dmbt1 overproduction, likely reflecting host compensatory mechanisms.^131^ Fructose is another nutrient part or the WD, and Montrose et al. recently demonstrated, in both *Citrobacter rodentium*-induced colitis and IL10^−/−^ models, that a high fructose diet is sufficient to worsen intestinal inflammation and damage the integrity of the gut barrier. A reduction of the mucus layer thickness associated with bacterial colonization was observed, demonstrating that numerous dietary factors are acting in combination to regulate the host/microbiota interactions at the mucosal surface.^132^

### Fiber-free diet

Studies indeed demonstrated that a fiber-depleted diet participates in chronic inflammatory diseases through modulation of microbiota composition and function.^133,134^ For example, mice fed a low-fat low-fiber diet exhibit increased body weight and altered metabolism compared to mice fed a fiber-rich diet.^135^ Other studies demonstrated that supplementation with oligofructose, a chicory inulin-type fructan, is sufficient to improve metabolic parameters altered by a HFD.^136–139^

Desai et al. elegantly demonstrated that fiber deprivation altered the intestinal microbiota in a way that promoted degradation of the colonic mucus barrier and enhanced pathogen expansion, exacerbating susceptibility to colitis.^140^ Using a synthetic minimal microbiota, they demonstrated that, in the absence of fiber, the gut microbiota shifted towards mucin degraders to fulfill its nutritional needs. Consequently, bacteria such as *A. muciniphila* or *B. thetaiotaomicron*, erode the colonic mucus barrier, hence enabling epithelium colonization by pathogenic bacteria, as demonstrated using the *Citrobacter rodentium* infection model. In 2016, Sonnenburg et al. demonstrated, in a mouse model, that dysbiosis and associated disorders induced by a fiber-deprived diet are reversible within a generation, but became non-reversible after multiple generations.^141^ Hence, a fiber-rich diet appears as an effective way to reinforce the intestinal barrier *via* its beneficial impact on mucosa-associated microbiota.

### Emulsifiers

Select food additives appear to play an important role in regulating mucus-associated microbiota composition and function. It has been hypothesized that emulsifiers, which are added to most processed foods to improve texture and extend shelf life, might have contributed to the rapid post–mid-20th century increase in the incidence of chronic inflammatory diseases.^142,143^ Investigation of this hypothesis demonstrated that dietary emulsifiers can indeed detrimentally impact the intestinal microbiota in a way that drives chronic inflammatory diseases. In wild-type mice, bacteria were only rarely observed within 10 µm of the epithelium, and the average closest bacteria detected over multiple high-powered fields was about 25 µm from the epithelium.^50^ In contrast, in mice fed with dietary emulsifying agents CMC and P80, bacteria could be found in direct contact with the epithelium, and the average distance of the closest bacteria per field was <10 µm.^50^ Such effects of emulsifier exposure on the microbiota were associated with the development of chronic colitis in susceptible mice, while wild-type mice developed chronic low-grade intestinal inflammation and metabolic deregulations. Importantly, microbiota/epithelium distance inversely correlated with the extent of intestinal inflammation, supporting the central and direct role played by mucus penetrating bacteria in emulsifier-induced promotion of chronic intestinal inflammation.^50^ It was also reported that in gnotobiotic mice colonized with a highly restricted microbiota comprised of only eight bacteria (namely “Altered Schaedler Flora”, ASF), emulsifier consumption was not sufficient to induce microbiota encroachment, intestinal inflammation, nor metabolism alteration. This thus suggested that a complex microbiota containing specific species infiltrating the mucus layer is required for the detrimental effect of emulsifiers.^144^ In more recent work, pathobiont colonization of ASF mice was observed to be sufficient to make the animals susceptible to microbiota encroachment and chronic intestinal inflammation induced by emulsifier consumption.^145^ Hence, this demonstrates that select bacteria are needed to mediate the detrimental effect of emulsifier exposure through their encroachment within the mucus layer and subsequent promotion of chronic intestinal inflammation. Further research is now warranted to identify these mucus invaders in both animal and human models.

## Mucosa-associated Microbiota—Opening of therapeutic avenues?

As detailed above, there is now evidence that mucosa-associated microbiota is crucial for host-bacteria interactions, which may open the door for therapeutic strategies in the coming years. On one hand, some commensals, such as *Akkermansia muciniphila*, may help to maintain mucosal integrity through a probiotic-type mechanism. On another hand, microbiota encroachment was reported to associate with an array of poor health outcomes, with the observation that a reduced epithelium-microbiota distance correlates with the severity of intestinal inflammation in mice and dysglycemia in human.^50,51^ While any causal link between microbiota encroachment and chronic inflammatory diseases remains to be studied, this underscores the need for novel approaches to target and inhibit encroachment of deleterious bacteria (Fig. 1).

### Probiotic approach: the example of *Akkermansia muciniphila*

Isolated in 2004 by Derrien et al.,^146^ *A. muciniphila* is a mucin-degrading bacterium and one of the most described commensal bacterium residing in the intestinal mucus layer.^147,148^ Its abundance is positively associated with metabolic health, as elegantly demonstrated by the work of De Vos’ and Cani’s teams.^149–151^ Everard et al. for example reported that mucus thickness is reduced by half in diet-induced obese mice, a phenomenon that can be prevented by *A. muciniphila* treatment.^149^ It was also reported that this bacterium can increase the number of regulatory T cells and goblet cells, as well as the expression of the antimicrobial Reg3 γ peptide, in the intestine.^152–154^ In vitro, *A. muciniphila* adheres to the intestinal epithelium and strengthens enterocyte monolayer integrity.^155^ More recently, a single protein, called Amuc_1100, was sufficient to improve gut barrier and partly recapitulate the previously observed beneficial effects in vivo.^156,157^ Moreover, it was reported that treatment with *A. muciniphila*-derived extracellular vesicles could enhance intestinal tight junction function, reduce body weight gain and improve glucose homeostasis in obese mice.^158^ Plovier et al. demonstrated that pasteurization of this bacterium did not affect its beneficial effects, but even potentiated it.^157,159^ Finally, in a human pilot study, pasteurized *A. muciniphila* tended to decrease fat-mass gain and hip circumference, as well as improved insulin sensitivity, total cholesterol and blood markers of liver dysfunction, inflammation and endotoxemia in a cohort of overweight human volunteers.^120^ Altogether, these data elegantly support the therapeutic interest of this bacterium able to colonize the mucosa.^149,160,161^

### Interleukine-33 (IL-33)

IL-33 belongs to the IL-1 cytokine family, binds the ST2 receptor, and is involved in various cellular pathways, including cytokine secretion, epithelial repair process and cell replication and survival.^162–165^ IL-33 appears as a crucial amplifier of the mucosal and systemic innate and acquired immune response.^166,167^ Interestingly, IL-33^−/−^ mice displayed a dysbiosis and a decrease in Paneth and goblet cells that associated with an increased susceptibility to colitis.^168–170^ In a mouse model of chronic colitis, IL-33 administration led to disease improvement and increased mucin production.^163,168,171–174^ Several publications demonstrated that IL-33 plays a role in preventing encroachment by pathogens such as helminths,^175–178^ *Clostridium difficile*,^174^ *Salmonella* Typhimurium^179^ and *H. pylori*.^180^ Thus, IL-33 emerges as a central actor in regulating the host/microbiota interactions at the mucosal surface. However, caution appears warranted, as some studies reported a colitogenic impact of IL-33. In the DSS-induced colitis model, IL-33 administration was found to exacerbate intestinal inflammation, while IL-33KO mice were protected against colitis.^181–183^ Hence, while this cytokine could be a therapeutic tool to prevent microbiota encroachment, its exact role in modulating the host/microbiota relationship remains to be fully elucidated.

### Mucosal immunization

It was recently hypothesized that the mucosal adaptive immune system, in close contact with the microbiota at the mucosal side, can be used to prevent microbiota encroachment by excluding motile bacteria from the mucus layer through targeted anti-flagellin response. This idea stemmed from a study revealing that flagellum appendage is central to the ability of bacteria to penetrate the colonic mucus layer,^71^ and from recent observations indicating that purified anti-flagellin antibody can rapidly shut down flagellin expression, and thereby bacterial motility.^184^ Based on these findings, it was speculated that flagellin immunization might result in lower levels of bioactive flagellin in a way that will prevent microbiota encroachment. Mice immunized with purified flagellin displayed strong fecal anti-flagellin IgG and IgA responses that associate with reduced expression of flagellin by the intestinal microbiota. Moreover, flagellin immunization was sufficient to increase the distance separating the microbiota from the epithelium in a way that correlated with protection against colitis and diet-induced obesity.^184^ Overall, these data support the concept that vaccination strategies aiming to prevent microbiota encroachment might protect against, or perhaps even treat, chronic inflammatory diseases with a microbiota component.

## Conclusion

Multiple lines of evidence point to the importance of mucosa-associated microbiota in health and diseases. Additional research is warranted to characterize this “hidden” ecosystem, and uncover mechanisms by which it can promote health as well as inflammatory and metabolic disorders. To date, most microbiota studies have relied on analyzing microbiota composition *via* 16S rRNA gene sequencing of fecal material. While the mucosa-associated microbiota is of central importance, its study requires access to intestinal biopsies combined with molecular/culturomic approaches. Moreover, the causal link between microbiota encroachment and the promotion of chronic inflammatory diseases remains to be fully elucidated. Recently developed approaches allowing to specifically study mucus-associated microbiota should help in this endeavor.^185,186^ Besides its composition, identification of gene expression by this specific community also appears warranted in order to understand mechanisms by which select microbiota members colonize this unique niche in a way that induce chronic intestinal inflammation. Hence, while this mucosal microbiology field of research is just emerging, it holds exciting promises for the prevention and/or treatment of chronic inflammatory diseases!
